# Somatostatin receptor subtypes 2 and 5 are associated with better survival in operable hepatitis B-related hepatocellular carcinoma following octreotide long-acting release treatment

**DOI:** 10.3892/ol.2013.1435

**Published:** 2013-07-01

**Authors:** YAO LIU, LI JIANG, YI MU

**Affiliations:** 1 Department of Pediatric Surgery, National Center for Cardiovascular Disease and Fuwei Hospital, Chinese Acadamy of Medical Sciences, Peking Union Medical College, Beijing 100037, P.R. China; 2 Department of Hepatobiliary Surgery, Beijing Ditan Hospital, Capital Medical University, Beijing 100015, P.R. China

**Keywords:** hepatocellular carcinoma, somatostatin receptor, survival, octreotide long-acting release, quantitative polymerase chain reaction

## Abstract

Liver resections for hepatocellular carcinoma (HCC) in cirrhotic livers are associated with early recurrence and poor survival. Somatostatin analogues (SSAs) have been reported to inhibit cell proliferation by interacting with specific somatostatin receptors (SSTRs) 2 and 5. The present study investigated whether SSTR expression in HCC was associated with the clinical outcome following octreotide long-acting release (LAR) treatment. Paired tumor and cirrhotic liver samples were obtained following a liver resection from 99 patients with stage I–II HCC and HBV-related cirrhosis. The expression of SSTR2 and 5 was assessed using quantitative (q)PCR and immunohistochemistry. The patients were classified into two groups, the high expression (n=47) and low expression (n=52) groups, based on the gene expression levels. The clinicopathological data and survival results of the two groups were compared. When compared with the surrounding cirrhotic tissue, the SSTR2 and 5 mRNA levels were significantly decreased in the HCC tissue. There were no significant differences between the groups with respect to the baseline characteristics. The tumor recurrence rate was significantly lower in the high expression group compared with that of the low expression group (63.83% vs. 82.69%; P=0.033). The 1-, 3- and 5-year disease-free and overall survival rates of the high expression group were 97, 89 and 71% and 98, 89 and 74%, respectively. The survival time of the members of the high expression group was longer compared with that of the low expression group. The multivariate analysis revealed that the TNM-7 stage and SSTR2 expression were independent prognostic factors for survival. In conclusion, SSTR mRNA expression correlated with survival in patients with early-stage hepatitis B virus (HBV)-related HCC who were treated with octreotide LAR following surgery. The inhibitory effects of SSAs on tumor growth may be mediated by SSTR expression.

## Introduction

Hepatocellular carcinoma (HCC) is one of the most serious complications of liver cirrhosis and the third most lethal cancer worldwide (1). HCC is particularly frequent in Asia due to a high prevalence of chronic hepatitis B virus (HBV) infection (2). Radical hepatic resection remains the only potentially curative treatment for early-stage HCC (3,4). However, until recently, the results have been disappointing (5,6). Preventing the post-operative recurrence and improving the long-term survival in HCC patients following a hepatectomy are issues that require urgent investigation.

Octreotide is an octapeptide that pharmacologically mimics natural somatostatin and has displayed regulatory or suppressive effects against various tumors (7). Somatostatin is believed to act via somatostatin receptors (SSTRs) that are expressed by responsive tumors. In particular, octreotide has a high affinity to SSTR subtypes 2 and 5. The expression of SSTRs in HCC tissue has been identified (8,9). In addition, the results of two studies using SSTR scintigraphy suggest that 40–50% of HCC cases express SSTRs *in vitro* and *in vivo*(10,11). This suggests that octreotide may be a valuable and promising approach for HCC treatment.

However, studies have been performed to verify the anti-tumor effects of octreotide long-acting release (LAR) treatment on advanced HCC, providing controversial results. Following the publication of the preliminary study by Kouroumalis *et al*(14), three randomized placebo-controlled studies have reported a significant survival benefit for long-acting octreotide compared with no treatment in patients with advanced HCC (11,15,16). Nevertheless, in the other five trials, no survival benefit was observed compared with the placebo for long-acting octreotide (12,13,17–19). In none of the aforementioned studies was an accurate estimation of SSTR gene expression in the tumors investigated, knowledge of which is critical if the therapeutic effects of somatostatin analogues (SSAs) are to be exploited.

In an attempt to obtain sufficient material for the pathological diagnosis and gene expression analysis, the present study was performed in patients with resectable early-stage HCC. The present study aimed to evaluate SSTR2 and 5 mRNA expression in a large number of surgically removed HCC tissues by quantifying specific PCR products with an accurate quantitative (q)PCR method. Furthermore, SSTR2 and 5 mRNA expression was also quantified in paired liver cirrhosis tissues to evaluate the expression of SSTR2 and 5 in tumor and cirrhosis tissues. The possibility of using SSTR expression as an efficacy predictor in HCC patients that are treated with octreotide LAR was further tested.

## Patients and methods

### Patients and samples

Tissue specimens of HCC and cirrhotic liver were randomly obtained from 99 patients who underwent curative liver resection at the Department of Hepatobiliary Surgery (Beijing Ditan Hospital, Capital Medical University, Beijing, China) between 2001 and 2004. HCC with underlying HBV-related cirrhosis was diagnosed by histological examination. No other treatment was administered prior to the surgery. Immediately after the hepatic resection for HCC, the tumor and surrounding cirrhotic liver tissue samples (≥1 cm away from the edge of tumor) were harvested and placed in liquid nitrogen until the RNA extraction procedure. The tissues that were used in the immunohistochemical studies were obtained from the same patients. The patient group consisted of 75 males and 24 females (average age, 58.2 years; range, 28–83 years). The clinicopathological records of these patients included gender ratio, age, location, maximum tumor size, serum α-fetoprotein level, serum HBV DNA level, tumor differentiation and staging. Tumor differentiation was defined according to the Edmondson grading system (20). Tumor staging was defined according to the 7th edition of the Union for International Cancer Control (UICC) TNM staging system (Table I) (21). Prior to starting the study, ethical approval was obtained from the ethical committee of Beijing Ditan Hospital. Informed consent was obtained from each patient who underwent octreotide treatment.

### Adjuvant therapy and follow-up

At one day post-surgery, all the patients were administered 20 mg octreotide LAR (Sandostatin LAR; Novartis-Pharma, Beijing, China) through a deep intramuscular injection every 4 weeks. The planned duration of treatment was 12 months in the absence of disease progression and unacceptable toxicity. All patients were observed prospectively for post-operative recurrence with assessment using serum α-fetoprotein levels, chest x-rays and ultrasonography or computed tomography 1 month after the operation and every 3 months thereafter. The patients with recurrence were administered a multimodality therapy, including a second liver resection, radiofrequency ablation or transarterial chemoembolization (TACE). The treatment decision was based on the pattern of recurrence and liver function reserve. Full follow-up data was recorded for all the patients until July 15, 2011.

### RNA extraction and cDNA synthesis

Total RNA was isolated from the tumor and paired cirrhotic tissues using TRIzol reagent (Invitrogen, Carlsbad, CA, USA). The concentration and purity of the RNA samples were determined using spectrophotometry (BioPhotometer; Eppendorf, Hamburg, Germany) and quantified by measuring the absorbance at OD 260. RNA quality was assessed by agarose gel electrophoresis. Following this, the RNA from each sample was reverse-transcribed to cDNA using the SuperScript III First-Strand Synthesis System for RT-PCR (Invitrogen) according to the manufacturer’s instructions.

### Synthesis of primers

Using the Primer 3 Analysis Software, version 1.1.0 (simgene.com, Whitehead Institute for Biomedical Research, Cambridge, MA, USA), the primer sequences were selected to optimally hybridize and amplify the target cDNA for the qPCR assay. To avoid amplifying the contaminating genomic DNA, the primers were designed so that each PCR product covered at least one intron. The target gene primers sequences used were as follows: SSTR2 forward, 5′-GTC CTC TGC TTG GTC AAG GTG-3′ and reverse, 5′-TGG TCT CAT TCA GCC GGG ATT-3′; and SSTR5 forward, 5′-GCC TGG GTC CTG TCT CTG TG-3′ and reverse, 5′-TAC CGC CCT CCT GCA CGT-3′. A housekeeping gene was used as an internal inference to avoid errors arising from the various efficiencies of the amplification. The primer sequences for GAPDH were forward, 5′-AGC CAC ATC GCT CAG ACA C-3′ and reverse, 5′-GCC CAA TAC GAC CAA ATC C-3′.

### qPCR

qPCR was performed in an ABI Prism 7500 Sequence Detection System (Applied Biosystems, Foster City, CA, USA). The reactions were performed in a 20-μl mixture containing 10 μl SYBR Green PCR Master Mix, 1 μl cDNA Template, 0.5 μl forward primer, 0.5 μl reverse primer and 8 μl ddH_2_O. The PCR thermal profile was 2 min at 50ºC followed by 10 min at 95ºC and 40 amplification cycles at 95ºC for 15 sec and 61ºC for 60 sec. The expression of the target genes was normalized using GADPH as an endogenous control and a sample number of 1,001 as a calibrator to correct for differences in the amount of total RNA added to each reaction. The cycle threshold (Ct) values were analyzed using the SDS 1.4 software (ABI Prism 7500, SDS User Bulletin; Applied Biosystems) and the relative mRNA levels were calculated using the comparative Ct method. The relative quantification (RQ) values were used to compare the gene expression levels between the various samples. Each sample was amplified in triplicate to obtain an average Ct value. A reaction without the cDNA templates was used as a negative control.

### Histopathological studies

Immunohistochemical evaluations were performed using the standard procedure. The specimens were fixed in 10% neutral-buffered formalin for at least 12 h, embedded in paraffin, sectioned (4-μm thick) and processed for immunohistochemistry. For SSTR2 and 5 expression, the section was incubated with anti-SSTR2 and -SSTR5 rabbit polyclonal antibodies (ab9550 and ab28618, respectively; Abcam Inc., Cambridge, MA, USA) with a dilution of 1:100 and 1:200, respectively. The negative control slides were treated with PBS solution as the first antibody under equivalent conditions. For the secondary developing reagents, a labeled streptavidin-biotin kit (Shenzhen Jingmei Biology Engineering Co. Ltd., Shenzhen, China) was used. The slides were developed using diaminobenzaminidine and counterstained with hematoxylin. A proportion score (PS) was assigned using the following system: 0, ≤10%; 1, 11–25%; 2, 26–50% and 3, >50%; and a staining intensity score (IS) was assigned as: 0, none; 1+, weak, 2+, intermediate and 3+, strong. A final total score (TS) was obtained from the sum of the PS and IS in at least three slides for each enrolled subject (22).

### Statistical analysis

The SPSS 15.0 program (SPSS, Inc., Chicago, IL, USA) was used for the statistical analyses. The results are expressed as the mean ± SD. The independent t-test was used to compare the relative gene copy number between the cirrhosis and HCC specimens. The χ^2^ test for the categorical outcome was used to compare the clinicopathological differences between the defined groups. The Pearson correlation coefficient was calculated to test the associations between the continuous variables. The Kaplan-Meier product-limit method was used to generate survival curves and the differences between the cohorts were tested using log-rank statistics. Cox’s proportional hazards regression model was used to analyze the independent prognostic factors. P<0.05 was considered to indicate a statistically significant difference. In the present exploratory study, no adjustments were made for multiple comparisons.

## Results

### Relative SSTR2 and 5 mRNA levels in HCC and paired cirrhosis

mRNA from SSTR2 and 5 was detected in all the HCC and surrounding cirrhotic liver tissues. qPCR analysis revealed that SSTR 2 and 5 were expressed at a lower level in the HCC tissues (1.76±0.92; 95% CI, 1.54–1.99; and 5.36±1.70; 95% CI, 4.94–5.77, respectively) compared with the paired surrounding cirrhotic liver tissues (3.46±1.45; 95% CI, 3.10–3.81; and 7.25±3.77; 95% CI, 6.32–8.18, respectively; t-value of HCC and cirrhotic tissues, 8.00 and 3.72, respectively; P<0.05).

### Expression of SSTR2 and 5 proteins in HCC and paired cirrhosis

**Figure 1:**
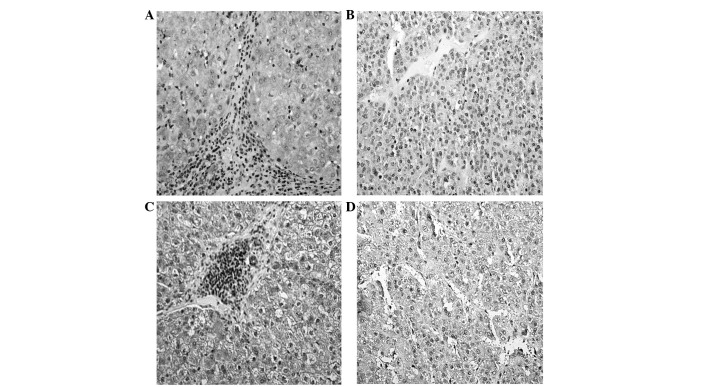
Immunohistochemical staining of SSTR2 and 5 in (A and C) non-tumorous tissues and (B and D) HCC. SSTR2 protein located in the cytoplasm of (A) cirrhotic liver and (B) HCC cells. SSTR5 was also expressed in the cytoplasm of (C) cirrhotic liver and (D) HCC cells. In the cirrhotic liver tissues, a higher intensity of immunostaining was observed compared with the HCC tissues. Original magnification, ×400. SSTR, somatostatin receptor; HCC, hepatocellular carcinoma.

An IHC analysis was performed in order to locate SSTR2 and 5 protein expression in HCC. Positive immunostaining for SSTR2 and 5 was localized primarily to the cell membrane and cytoplasm of the cirrhotic liver cells and the tumor cells (Fig. 1). SSTR2 expression in the tumor tissue was lower than in the surrounding cirrhotic liver tissues at a positive rate of 59.6% (59 of 99 cases) vs. 90.9% (90 of 99 cases; χ^2^, 26.06; P <0.001). SSTR5 was the most commonly expressed receptor subtype, with a positive expression rate in the HCC tissue of 95.5%, identical to that of the paired cirrhotic tissue. Furthermore, in the HCC samples, SSTR2 expression detected by qPCR and immunohistochemistry showed a strong correlation (r=0.312; P=0.002), as did SSTR5 expression (r=0.384; P=0.001).

### Baseline characteristics of the low and high expression groups

As shown previously, the mean SSTR2 expression in the HCC samples was 1.76 relative copies. The tumor samples that expressed SSTR2 above this cutoff were defined as having high SSTR2 expression and those that expressed SSTR2 below this level were defined as having low expression. Likewise, a cutoff point for SSTR5 was defined at 5.36 relative copies based upon the mean SSTR5 expression in the HCC tissues. When these defined strata were applied to the series of 99 HCC specimens, two groups were categorized based on the co-expression of SSTR2 and 5. A total of 52 (53%) patients had tumors with low SSTR2 and 5 expression and 47 (47%) had tumors with high SSTR 2 and/or 5 expression. Table II shows the baseline characteristics of the two groups. No differences in the clinical characteristics, including age, gender, histological grade and stage, were observed between the groups.

### Prognostic roles

**Figure 2:**
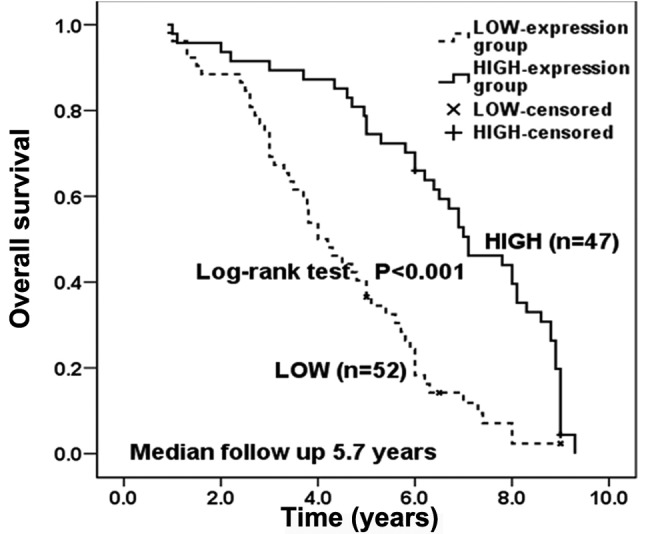
Cumulative overall survival curves of the patients who underwent post-operative octreotide LAR therapy in the low and high expression groups. LAR, long-acting release.

The drug was well tolerated, as 97 (98%) of 99 patients were administered LAR octreotide for the full 12 months (13 doses). A total of 10 patients from the low expression group and 17 from the high expression group did not experience recurrence during follow-up. With the exception of three patients who survived, the remaining 24 patients succumbed; the non-tumorous causes of mortality were liver failure (9 patients), heart disease (4 patients), cerebral vascular accident (4 patients), car accident (3 patients) and miscellaneous (4 patients). The overall survival times were significantly longer in the high expression group than the low expression group (Fig. 2). The median overall survival was 7.1 years (95% CI, 5.9–8.3) in the high expression group and 4.0 years (95% CI, 3.3–4.7) in the low expression group. The cumulative 1-, 3-, and 5-year overall survival rates were 98, 89 and 74%, respectively, for the high expression group and 96, 69 and 36%, respectively, for the low expression group.

**Figure 3:**
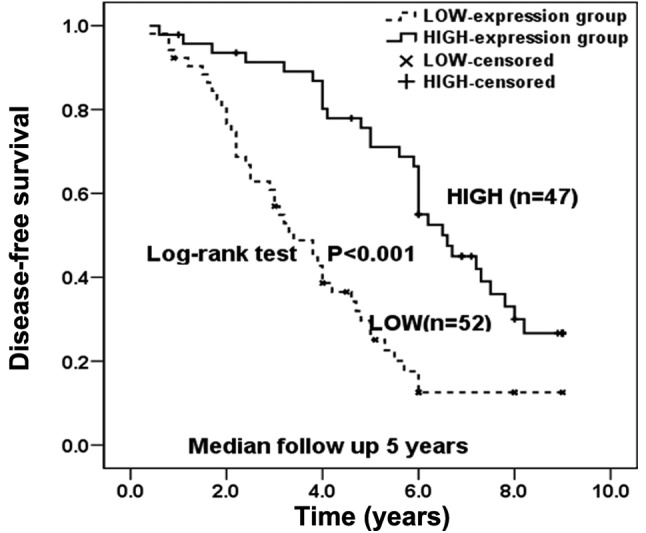
Disease-free survival curves of the patients who underwent post-operative octreotide LAR therapy in the low and high expression groups. LAR, long-acting release.

The disease-free survival results were also significantly improved in the high expression group compared with the low expression group (Fig. 3). The median disease-free survival was 6.6 years (95%CI, 5.2–8.0) in the high expression group and 3.4 years (95% CI, 2.5–4.3) in the low expression group. The cumulative 1-, 3-, and 5-year disease-free survival rates were 97, 89 and 71%, respectively, for the high expression group and 90, 55 and 25%, respectively, for the low expression group. The overall incidence of tumor recurrence was significantly lower in the high expression group than in the low expression group (63.83 vs. 82.69%; χ^2^=4.54; P=0.033).

To evaluate the potential of using SSTR expression in determining the post-operative prognosis of HCC patients, a multivariate analysis was conducted using a Cox proportional hazard regression model. All the clinicopathological characteristics and the SSTR2 and 5 mRNA levels were included in the model. The Cox multivariate analysis revealed that SSTR2 expression levels may be used as an independent prognostic marker for operable and TNM-7 stage HCC (Tables III and IV).

## Discussion

The expression of SSTRs in tumor cells allows new possibilities for the treatment and diagnosis of patients with HCC. Five subtypes of SSTRs, SSTR1–5, have been cloned and belong to a distinct group within the super family of G-protein-coupled receptors with seven transmembrane regions (23). As measured by autoradiography, 41% of HCCs have been shown to be SSTR-positive, predominantly subtype 2, which is consistent with the study by Bläker’s *et al*(9,11). In the study, HCCs displayed differential, individual expression patterns, as well as variable SSTR expression levels. The overall expression rate of SSTR1, 2, 3, 4, and 5 was 46, 41, 64, 0 and 75%, respectively. SSTR occupancy represents the basis for *in vivo* tumor targeting, and is a significant consideration in determining the clinical efficacy of somatostatin therapy.

Pharmacological studies have already shown that SSA octreotide acts mainly via two SSTRs (SSTR2 and 5) expressed on responsive tumors (24). In the present study, qPCR was used to identify the differential SSTR expression profiles between HCC and the surrounding non-tumorous cirrhotic tissues. The present data revealed a wide range of SSTR2 and 5 expression in the tumor and cirrhosis samples. However, downregulation was noted in the HCC specimens. Similarly, Reynaert *et al* were able to demonstrate the presence of SSTRs in the majority of HCC and adjacent cirrhotic liver tissues using the PCR technique (8). In another study, Xie *et al* also identified that ~60% of HCCs expressed SSTRs, as well as the non-tumor cirrhotic liver tissues (25).

In the present study, the HCC specimens had a 1.95- and 1.35-fold reduction in SSTR2 and 5 mRNA levels, respectively, as compared with the adjacent cirrhotic liver tissues. Similar to this observation, Reynaert *et al* also identified that in two of six patients, the surrounding cirrhotic liver tissues expressed SSTR5 mRNA more clearly than the tumors of these patients. As they did not use a qPCR method, they were not able to draw firm conclusions with regard to the variation in mRNA expression (8). This observation corresponded with the findings made in pancreatic and colorectal cancer studies (26,31,32). In contrast to normal tissue or benign lesions, there is a loss of SSTR2 gene expression in pancreatic carcinoma and advanced colorectal cancer and their respective metastases (26,31–33). SSTR2 expression was selectively lost in 90% of the human pancreatic carcinomas and derived pancreatic cell lines. Reintroducing SSTR2 in human pancreatic cancer cells by stable expression resulted in a constitutive activation of SSTR2 and an inhibition of cell growth in the absence of an exogenous ligand. These effects resulted from an increased expression and secretion of the somatostatin ligand, thus leading to a negative autocrine loop. The negative feedback loop may also exist in liver cancer. Additionally, insulin-like growth factor-1 (IGF-1), which is produced by hepatocytes as an endocrine hormone, has been shown to play a pathogenic role in cancer, and octreotide has been shown to negatively control serum IGF-1 levels, possibly via SSTR2 and SSTR5, and a direct downregulation of IGF gene expression (35). Apoptosis has also been shown to be induced by SSTR2 in human pancreatic cancer cells expressing mutated p53 that were devoid of endogenous SSTR2, following the correction of the deficit by the expression of SSTR2 (36). The absence of SSTR2 and SSTR5 may explain the lack of local response to octreotide therapy in certain advanced liver cancers.

In the normal liver, hepatocytes and HSCs have been shown to be negative for all five SSTRs (8). During the preneoplastic stage leading to HCC, SSTR gene expression levels in liver tissues appear to be altered regularly (9,34). SSTR expression levels increase with the progress of disease conditions, i.e. from normal liver tissues, hepatitis, liver cirrhosis and reaching a peak in the precancerous lesions (25). Once HCC has developed, transcription of the SSTR gene does not increase further, supporting its major involvement in the preneoplastic stage. Downregulation of SSTR transcription may result in a loss of a tumor suppressive effect of SSTRs in human HCC.

Notably, the results of the present study were not compatible with the investigation performed by Xie *et al*(25), in which an overexpression of SSTRs was identified in HCC. The main reason for this was that in the study by Xie *et al,* the controlled cirrhotic liver and HCC tissues were not obtained from the same HCC patient, but from other patients with non-tumor liver cirrhosis. Furthermore, the present study also analyzed SSTR expression in non-tumor cirrhotic liver tissues from 10 cirrhotic patients with chronic hepatitis B and obtained the same results as Xie *et al* (data not shown).

Octreotide is an octapeptide that mimics natural somatostatin pharmacologically and possesses potent antineoplastic activity in several human cancers (7). The use of octreotide as a monotherapy has been observed to prolong survival in patients with unresectable HCC (11,14–16). Furthermore, in an animal study, nude mice were administered with octreotide for 35 days following a surgical removal of human HCC xenografts. Compared with the control group, octreotide at doses of 100 and 200 μg/kg/day significantly inhibited the growth rate of second primary tumors, decreased lung metastasis and prolonged the life span (27). Although other controlled trials have reported no survival benefit for long-acting octreotide compared with placebos (12,13,17–19), certain researchers believe it is possible that octreotide LAR may benefit a subgroup of patients whose tumors express high levels of SSTRs (19).

In the present study, to verify this hypothesis, the patients with HCC were divided into two defined groups according to the SSTR2 and 5 expression levels. The two groups were compared for clinicopathological data and survival results. The statistical analysis revealed that the patients in the two groups were of similar age and had a similar tumor morphology distribution. This observation corresponds to the findings observed by Bläker *et al*(9), where the expression of the SSTRs appeared to be independent of tumor stage and/or differentiation, which was also similar to the findings in gastrointestinal and pancreatic endocrine tumors (28).

The present study showed a significant difference in the survival rates between the low and high expression groups. The overall and disease-free survival outcomes were significantly improved in the high expression group compared with the low expression group. The multivariate model also revealed that SSTR2 expression levels may be used as an independent prognostic marker of HCC, as well as tumor TNM-7 stage. Similar results have been reported in other types of cancers, including neuroblastomas (29) and breast (30), pancreatic and colorectal cancers (31,32). The overexpression of SSTR2 has also displayed anti-tumor effects and significantly increased the sensitivity of SSTR treatment in a number of experimental SSTR-negative cancer cell lines and xenografts (33,34). Although this analysis requires validation from larger prospective series, SSTR gene expression levels, particularly those for SSTR2, appear to be viable molecular markers to appropriately select HCC patients for post-operative octreotide LAR therapy.

To the best of our knowledge, the present study is the first to investigate the association of SSTR gene expression levels with the long-term prognosis of patients with early-stage HCC undergoing post-operative octreotide LAR treatment. The downregulation of SSTR transcription may result in the loss of tumor suppression. SSTR mRNA expression was shown to correlate with survival in patients with early-stage HBV-related HCC who were treated with octreotide LAR. The inhibitory effects of SSAs on tumor growth may be mediated by SSTR expression.

## Figures and Tables

**Table I:** Seventh edition UICC TNM stage of HCC (2009).

Stage	Tumor	Node	Metastasis
I	T1	N0	M0
II	T2	N0	M0
IIIA	T3a	N0	M0
IIIB	T3b	N0	M0
IIIC	T4	N0	M0
IVA	Any T	N1	M0
IVB	Any T	Any N	M1

UICC, Union for International Cancer Control; HCC, hepatocellular carcinoma; T1, single tumor without vascular invasion; T2, single tumor with vascular invasion or multiple tumors of <5 cm; T3a, multiple tumors of >5cm; T3b, single tumor or multiple tumors of any size involving a major branch of the portal or hepatic veins; T4, tumors with direct invasion of adjacent organs other than the gallbladder, or perforation of the visceral peritoneum; N1, regional lymph node metastasis; M1, distant metastasis.

**Table II:** Descriptive characteristics of the low and high expression groups.

	Group, n (%)			
			
Characteristics	Low, (n=52)	High, (n=47)	χ^2^	P-value
Gender			0.277	0.599
Male	39 (75)	36 (77)		
Female	13 (25)	11 (23)		
Age, years			0.015	0.903
<55	13 (25)	15 (32)		
≥55	39 (75)	32 (68)		
Child-Pugh grade			0.020	0.886
Grade A	15 (29)	18 (38)		
Grade B	37 (71)	29 (62)		
AFP(ng/ml)			0.379	0.538
≥400	15 (29)	12 (26)		
<400	37 (61)	35 (74)		
HBV-DNA(copies/ml)			0.218	0.640
≥500	30 (58)	26 (55)		
<500	22 (42)	21 (45)		
Location			0.707	0.400
Left Lobe	22 (42)	14 (30)		
Right Lobe	30 (58)	33 (70)		
Tumor size (cm)			0.054	0.817
≤3	36 (69)	29 (62)		
>3	16 (31)	18 (38)		
Microvascular invasion			0.379	0.538
Present	15 (29)	12 (26)		
Absent	37 (71)	35 (74)		
Edmondson-Steiner grade			0.244	0.621
I–II	19 (37)	17 (36)		
III–IV	33 (63)	30 (64)		
TNM-7 stage			0.347	0.556
I	31 (60)	30 (64)		
II	21 (40)	17 (36)		

AFP, α-fetoprotein; HBV, hepatitis B virus.

**Table III:** Univariate and multivariate analysis of overall survival of patients with HCC.

	Univariate analysis			Multivariate analysis		
		
Variable	P-value	RR	95% CI	P-value	RR	95% CI
Gender (male vs female)	0.407	0.808	0.487–1.338			
Age, years (≥55 vs. <55)	0.831	0.957	0.636–1.438			
Child-Pugh grade (A vs. B)	0.976	0.993	0.644–1.532			
AFP, ng/ml (>400 vs. <400)	0.952	0.988	0.657–1.485			
Location (left vs. right)	0.270	0.789	0.518–1.202			
Tumor size, cm (>3 vs. ≤3)	0.238	1.285	0.847–1.951			
HBV-DNA, copies/ml (≥500 vs. <500)	0.002	1.936	1.265–2.962	0.321	1.271	0.792–2.040
Microvascular invasion (present vs. absent)	0.000	2.410	1.539–3.773	0.094	1.506	0.933–2.431
Edmondson-Steiner-grade (I–II vs. III–IV)	0.002	1.986	1.287–3.064	0.282	1.328	0.792–2.226
TNM-7 stage (I vs. II)	0.000	2.579	1.692–3.932	0.002	2.303	1.499–3.539
SSTR-2 expression	0.000	0.472	0.372–0.598	0.000	0.531	0.372–0.758
SSTR-5 expression	0.000	0.754	0.663–0.857	0.682	0.965	0.814–1.144

HCC, hepatocellular carcinoma; CI, confidence interval; RR, relative risk, AFP, α-fetoprotein HBV, hepatitis B virus; SSTR, somatostatin receptor.

**Table IV:** Univariate and multivariate analysis of disease-free survival for patients with HCC.

	Univariate analysis			Multivariate analysis		
		
Variable	P-value	RR	95% CI	P-value	RR	95% CI
Gender (male vs. female)	0.488	0.817	0.462–1.446			
Age, years (≥55 vs. <55)	0.270	1.299	0.816–2.070			
Child-Pugh grade (A vs. B)	0.737	0.920	0.566–1.496			
AFP, ng/ml (>400 vs. <400)	0.879	1.037	0.651–1.652			
Location (left vs. right)	0.294	0.776	0.482–1.247			
Tumor size, cm (>3 vs ≤3)	0.321	1.268	0.793–2.025			
HBV-DNA, copies/ml (≥500 vs. <500)	0.007	1.920	1.198–3.076	0.131	1.494	0.888–2.514
Microvascular invasion (present vs. absent)	0.006	1.972	1.216–3.196	0.816	1.065	0.626–1.812
Edmondson-Steiner-grade (I–II vs. III–IV)	0.030	1.691	1.053–2.715	0.953	1.017	0.581–1.779
TNM-7 stage (I vs. II)	0.000	3.263	2.011–5.297	0.000	3.158	1.858–5.367
SSTR-2 expression	0.000	0.475	0.362–0.624	0.004	0.542	0.359–0.818
SSTR-5 expression	0.000	0.708	0.607–0.827	0.318	0.902	0.737–1.105

HCC, hepatocellular carcinoma; CI, confidence interval; RR, relative risk, AFP, α-fetoprotein HBV, hepatitis B virus; SSTR, somatostatin receptor.
